# Effect of socioeconomic status on the physical and mental health of the elderly: the mediating effect of social participation

**DOI:** 10.1186/s12889-022-13062-7

**Published:** 2022-03-29

**Authors:** Yunfan Zhang, Dai Su, Yingchun Chen, Min Tan, Xinlin Chen

**Affiliations:** 1 Department of Health Management, School of Medicine and Health Management, Tongji Medical College, Huazhong University of Science and Technology, Wuhan, 430030 China; 2 Research Centre for Rural Health Service, Key Research Institute of Humanities & Social Sciences of Hubei Provincial Department of Education, Wuhan, China; 3 First Affiliated Hospital of Guangzhou Medical University, Guangzhou, China

**Keywords:** Socioeconomic status, Mediation effect, Social participation, Elderly health

## Abstract

**Background:**

Previous studies have demonstrated the effect of socioeconomic status on the health status of the elderly. Nevertheless, the specific dimensions of the effect and the mechanism await further investigation. In this study, socioeconomic status was divided into three dimensions and we used social participation as the mediation variable to investigate the specific path of effect.

**Methods:**

Using the 2018 Waves of Chinese Longitudinal Healthy Longevity Survey (CLHLS) dataset, a total of 10,197 effective samples of the elderly over 65 years old were screened out. Socioeconomic status included income, education level, and main occupation before retirement. The physical health and mental health of the elderly was measured by the Instrumental Activities of Daily Living Scale and the Minimum Mental State Examination, respectively. The social participation of the elderly was the mediation variable, including group exercise, organized social activities and interacting with friends. Omnibus mediation effect analysis was adopted to examine the mediation effect and mediation analysis was completed using the SPSS PROCESS program.

**Results:**

First, the results showed that when the income gap between the elderly reached a certain level, there was a significant difference in health status. Significant differences existed in health status amongst with different education levels. There was no sufficient evidence to show that occupation has a significant effect on the physical health. But when the dependent variable was mental health, the effect was significant. Second, group exercise mediated 64.11% (a_i_b = 0.24, 95% CI [0.17,0.3]) and up to 20.44% (a_i_b = 0.12, 95% CI [0.07,0.17]) of the disparity in physical and mental health due to income gap, respectively. And it could mediate the effect up to 56.30% (a_i_b = 0.62, 95% CI [0.52,0.73]) and 17.87% (a_i_b = 0.50, 95% CI [0.4,0.61]) of education on physical and mental health status, respectively. The proportion of relative mediation effect of occupation was up to 28.74% (a_i_b = 0.19, 95% CI [0.13,0.25]) on mental health. Interacting with friends mediated only on the path that the education affected the health status of the elderly. The proportion was up to 33.72% (a_i_b = 0.29, 95% CI [0.16,0.44]). The relative mediation effect of organized social activities on the health gap caused by income or education level gap was significant at some levels. The proportion was up to 21.20% (a_i_b = 0.33, 95% CI [0.26,0.4]).

**Conclusion:**

The SES of the elderly including relatively large income gap, different education levels and occupational categories could indeed have a significant effect on health status of the elderly, and the reason why this effect existed could be partly explained by the mediation effect of social participation. Policymakers should pay more attention to the social participation of the elderly.

## Background

Ageing is an important issue that cannot be ignored in modern Chinese society. The population aged 60 and over exceeds 260 million, accounting for 18.70%. The elderly population in China is expected to exceed 300 million and step from mild ageing into moderate ageing before 2025. As an important basis for ensuring the daily life and social activities of this large group, improving the health status of the elderly is a very critical issue. Actively coping with population ageing and improving the health status of the elderly has become a national strategy for every country. Accordingly, the factors affecting the physical and mental health of the elderly have always been the focus of scholars’ research. The theory of health production proposed by Grossman [1] indicates that health may be affected by health care, income level, lifestyle, education level, and living environment, amongst others. Amongst all these factors, socioeconomic status (SES) has received increased attention in recent years.

SES is defined as an individual or group’s position within a hierarchical social structure, reflecting the social class and status of different groups [2, 3]. It is a comprehensive indicator of income level, education level, occupational status, and wealth, and these resources may enable people to achieve certain goals [4]. It is also an important indicator to measure and predict people’s behaviour [5]. Research on the relationship between SES and health could be traced back to the 1950s. Most early studies have focused on the role of structural factors, i.e., SES affects personal health through health literacy, accessibility of medical services, and living environment [6]. In recent years, the influence of SES on health has been extended to lifestyle factors, social psychological factors, and other aspects. Marmot [7] found that SES affects health through social gradient, income, social exclusion, education, psychological status, and other factors. People with lower SES have lower autonomy to work, corresponding to more pressure and negative emotions. Cristine et al. [8] believed that people with adverse SES are more likely to fall into a negative environment, have negative emotions, and suffer from potential stress, all of which have a negative effect on health. SES also reportedly affects health through interaction across different factors [9], including exposure to environmental toxins, air and water pollution, or poor nutrition, as well as adverse health behaviours such as smoking, excessive alcohol intake, sleeping patterns, and physical inactivity [10–12]. Warr [13] believed that SES often affects individual health status through the joint action of local culture, neighbourhood environment, or social and psychological factors. The research above has revealed the close relationship of the SES with the physical and mental health of the elderly. However, most of them did not point out the clear influence path, and there was hardly any measurement and report on the mediation effect of the possible mediation variables.

The Anderson model [14, 15] indicates that predisposing factors and enabling factors affect health status through health behaviours. As an important element of predisposing factors, SES has also been shown to have a significant effect on health status. Health behaviours include personal self-care and health service utilisation. In self-care behaviours, the social participation has attracted more and more attention, which may play an important role in the process by which SES affects health status. At present, there is no accurate definition of social participation [16]. The social participation of the elderly is generally believed to be primarily reflected in three aspects. First, from the perspective of role intervention, it emphasises that elderly people play a meaningful social role in leisure or productive activities [17]. This definition reflects the identity and role attribution of the elderly in the process of social participation. Second, from the perspective of social interaction [18], it emphasises interaction with people other than spouses in formal or informal occasions [19]. This definition usually regards social communication and interaction as the core components of social participation of the elderly. Third, from the perspective of function exertion, social participation of the elderly is defined as their meaningful participation in social and productive activities [20] or as engaging in activities involving personal actions and contributions to others. Many studies have shown that social participation can significantly affect the physical and mental health of the elderly, and that more social participation can significantly reduce the risk of death or disability [21–24]. It is believed to be a crucial component of positive ageing [25]. Activities such as visiting relatives and friends or chatting with friends and relatives are related to longevity [26]. Some studies have also revealed that social participation can effectively reduce the risk of depression in the elderly, and that active social participation can significantly prevent and alleviate depression and improve their mental health [27–30]. At the same time, some scholars have found that factors such as income, occupation, and education can significantly affect the level of individual social participation [31, 32].

Therefore, based on the Anderson model, this study aimed to determine the effect mechanism of SES on the physical and mental health of the elderly, as well as to explore the mediation effect of social participation. Based on existing research results, the hypotheses of this article were as follows.

- Hypothesis1: Socioeconomic status was positively related to health status.
- Hypothesis1a: Annual household income of the elderly was positively related to health status.
- Hypothesis1b: Education level of the elderly was positively related to health status.
- Hypothesis1c: Main occupation before retirement of the elderly was related to health status.
- Hypothesis 2: Social participation mediated the effect of socioeconomic status on health status.
- Hypothesis 2a: Group exercise mediated the effect of socioeconomic status on health status.
- Hypothesis 2b: Organized social activities mediated the effect of socioeconomic status on health status.
- Hypothesis 2c: Interacting with friends mediated the effect of socioeconomic status on health status.

## Materials and methods

### Sample

The data for this study were obtained from the cross-sectional data of the ‘Chinese Longitudinal Healthy Longevity Survey (CLHLS)’ co-developed by the Chinese Center for Disease Control and Prevention and the Research Center for Healthy Ageing and Development of the National Institutes of Development of Peking University in 2018. Since 1998, the research center had randomly selected about half of the counties and cities in 23 provinces, cities, and autonomous regions in China for eight follow-up surveys, A total of 113,000 home visits were conducted, covering many research contents related to the family SES, daily living conditions, insurance, and health of the elderly aged over 65 [33, 34]. This study used the cross-sectional data from the latest seventh wave survey in 2018. Initially, 12,874 samples were included. After identifying and deleting duplicate values, missing values, and outliers with logical problems, 10,197 valid samples were obtained. The samples were well representative.

### Variables and instruments

#### SES

The SES of the elderly was measured using three variables, including annual household income, education level, and main occupation before retirement. Most people aged over 65 no longer had a fixed salary but were supported by their children after retirement, so the annual family income served as an indicator to measure the economic level of the elderly. This indicator has also been proven to be well representative [35]. Education levels ranged from illiteracy to senior and above. The classification of occupations was based on International Standard Classification of OECD (ISCO-88). Government staff and professional technicians were white-collar workers, and those engaged in business and service industry were blue-collar workers. The sample size of respondents engaged in agricultural was too large, so it was treated as a separate category in this study.

#### Health status

Instrumental Activities of Daily Living Scale (IADL) was used to measure the physical health of the elderly. IADL included eight indicators, such as visiting neighbours, shopping, cooking, washing clothes, and walking 1 km. The options for each indicator were assigned to ‘1 = needs a lot of help’, ‘2 = needs some help’, and ‘3 = no need for help’, with a higher score indicating better physical health. The mental health status of the elderly was measured with the results of the Minimum Mental State Examination (MMSE), which was developed by Folstein [36] in 1975. The cognitive scale used in this survey was slightly modified from the Li Ge scale for the elderly [37] and were calculated based on the respondents’ scores on all 24 questions, except C2-2 in section C (Competency test) of the questionnaire. The scale was modified as follows: (1) six time–place-oriented questions were reduced, and the elderly were not required to answer the name of their province (city), county (district), township/town (street), ‘what floor are we on now’, and other questions that were not closely related to their daily life; and (2) in the language test question, ‘say a complete sentence’ was changed to ‘the number of food names spoken in one minute’. The maximum score of this question was 7 points: 1 point for one food name, 2 points for two food names, and 7 points for seven or more food names. Thus, the total score was still 30 points. A higher scale score indicated better cognitive function. The Cronbach coefficient of the modified scale was 0.984 [38]. When calculating the MMSE score, this study adopted the MMSE calculation method of published articles and the cognitive function algorithm of the Chinese Elderly Health and Longevity Survey with the same CLHLS survey data [39].

#### Mediating variables

According to the definition of social participation by Bath and other scholars [40], the social participation of the elderly was divided into group exercise, organized social activities and interacting with friends. In the questionnaire of CLHLS, there were six questions related to the social participation of the elderly. Practising Tai Chi, square dance belonged to group exercise. Organised social activities was a separate item. Playing cards, playing mah-jongg and dropping in with friends belonged to interacting with friends. For every question, the answers ranged from 1 (never), 2 (not every month, but sometimes), 3 (not every week, but at least once a month), 4 (not every day, but at least once a week) and 5 (almost every day). The sum of the scores of each question under the corresponding dimension was the value of the variable. A higher score indicated greater social involvement deeper degree of social participation. Some scholars have used the similar method to measure social participation [41]. The Cronbach’s alpha coefficient of social participation in this study was 0.72.

#### Control variables

These variables included sociodemographic characteristics (including age, sex, and marital status), district, urban and rural areas, distance to the nearest medical institution, medical insurance and pension insurance status. The variable definition and assignment were shown in Table 1.

**Table 1:** Variable definition and assignment

Independent variable	Variable definition and assignment
Socioeconomic status	
Income	Annual household income. 0 = less than 10,000 yuan (Very low-income); 1 = 10,000-30,000 yuan (low-income);2 = 30,000-9000 yuan (medium income); 3 = 90,000 yuan and above (high income)
Education	The highest degree so far.0 = illiteracy;1 = primary;2 = junior;3 = senior and above
Occupation	Work before retirement.0 = agriculture;1 = governmental & professional;2 = commercial & service
Health status	
IADL	Value range:8-24 points
MMSE	Value range:0-30 points
Mediating variables	
Group exercise	Such as square dance/Tai chi etc.; Value range:3-15 points
Organized social activities	Such as volunteer etc.; Value range:1-5 points
Interact with friends	Such as play cards/mah-jongg etc.; Value range:2-10 points
Covariate	
Age	0 = Aged 75 or younger; 1 = 75-84 years old; 2 = 85-94 years old; 3 = Aged 95 or older
Gender	0 = male;1 = female
Residential area	0 = city;1 = town;2 = rural
Marital status	0 = married and living with a spouse;1 = separated (widowed or divorced)
District	0 = East;1 = Central;2 = West;
Accessibility of health services	The distance of the nearest hospital from home .0 = within 1 km; 1 = 1-3 km; 2 = up to 3 km;
Medical insurance	0 = no;1 = yes;
Pension insurance	0 = no;1 = yes;

### Methods

The characteristic distribution of the study participants was calculated using frequency for categorical variables, and means and standard deviations for numerical variables.

**Fig. 1:**
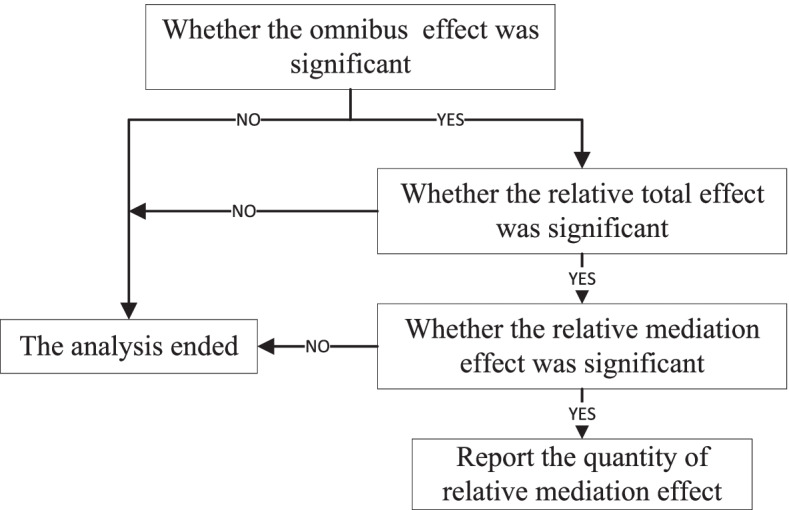
Processing flow for the omnibus mediation effect analysis

**Fig. 2:**
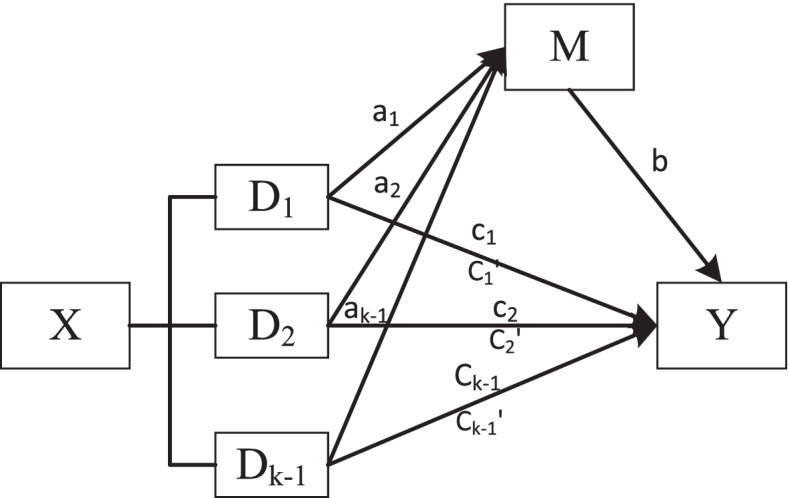
The mediation model with multicategorical independent variable(X: independent variable; Y: dependent variable; M: mediation variable; a_i_: the relative effects of the other k-1 levels, respectively, relative to the k level, on mediation variable; b: the association between mediation variable and dependent variable after controlling for covariates; c_i_: relative total effect of the k-1 levels, respectively, relative to the k level, on dependent variable after controlling for covariates; c’_i_: relative direct effects of the k-1 levels, respectively, relative to the k level, on dependent variable after controlling for mediation variable and covariates

On the choice of the test method for the mediation effect parameter, considering that the three independent variables in this study were multicategorical variables, which did not meet the requirements of variable distribution in the Sobel test, we chose the method of omnibus mediation effect analysis based on the research results of Preacher and Hayes [42, 43]. We set *k* dummy variables for every multicategorical independent variable. The mediation models require estimation of *k-1* direct and indirect effects. The convention proposed by Hayes and Preacher of referring to these as relative direct effects and relative indirect effects to acknowledge that the effects are quantified relative to a reference group was adopted. We completed the analysis in three steps. As shown in Fig.1, first, omnibus effect analysis was conducted, including the test of omnibus total effect, omnibus direct effect and omnibus mediation effect. If the omnibus effect was not significant, it meant that the *k-1* relative mediation effects did not exist, and the analysis ended. Otherwise, proceed to step 2. Second, we made sure that whether the relative total effect test of every independent variable on dependent variable was significant. Third, relative mediation effect was conducted, identifying which one or several of these had a significant relative mediation effect. And the corresponding result of relative mediation effect was reported. Referring to Fig. 2, that was a mediation model with multicategorical independent variable. The a_1_, a_2_, and a_k-1_ meant the relative effects of the other *k-1* levels, respectively, relative to the *k* level, on mediation variable. The b meat the association between mediation variable and dependent variable after controlling for covariates. The c_1_, c_2_, and c_k-1_ meant relative total effect of the k-1 levels, respectively, relative to the *k* level, on dependent variable after controlling for covariates. The c^’^_1_, c^’^_2_, and c^’^_k-1_ meant relative direct effects of the k-1 levels, respectively, relative to the *k* level, on dependent variable after controlling for mediation variable and covariates. The quantity of relative mediation effect equalled to |a_i_b/c_i_|.

All statistical analyses were completed using IBM SPSS Statistics version 26. We used SPSS macros PROCESS written by Andrew F. Hayes [44] to conduct the mediation effect analysis. Bootstrap sampling was set as 5000. The confidence interval was 95%. Relative indirect effects with confidence intervals that do not include zero show evidence of mediation.

## Results

### Characteristics of samples

Table 2 showed the demographic characteristics of the respondents. With respect to age group, there were 2402 (23.56%) respondents aged 75 or younger and 2826 (27.71%) were aged 95 or older. The proportion of each age group was relatively close. Of all the respondents, there were 4320 (42.37%) males and 5877 (57.63%) females. 4261 (41.79%) respondents married and living with spouse and the percentage of the respondents living alone (widowed or divorced) was 58.21%. More than 40% of the elderly lived in rural areas, and the number of elderly people living in city was 2606 (25.56%) and in town was 3297 (32.33%). More than half of the elderly came from eastern China. In terms of access to health services, 53.17% of the respondents lived within 1 km of the nearest health facility. However, there still had 1774 (17.40%) respondents lived more than 3 km of the nearest health facility. Most of the elderly people (87.69%) had medical insurance but only 29.37% of the respondents had pension insurance.

**Table 2:** The characteristics of sample (*N* = 10,197)

Variables	Category	N	Percentage (%)
**Age**	≤75	2402	23.56
	75-84	2518	24.69
	85-94	2451	24.04
	≥95	2826	27.71
**gender**	male	4320	42.37
	female	5877	57.63
**marital status**	married and living with a spouse	4261	41.79
	separated (widowed or divorced)	5936	58.21
**residential area**	city	2606	25.56
	town	3297	32.33
	rural	4294	42.11
**district**	east	5210	51.09
	middle	2686	26.34
	west	2301	22.57
**Accessibility of health services**	within 1 km	5422	53.17
	1-3 km	3001	29.43
	up to 3 km	1774	17.40
**Medical insurance**	no	1255	12.31
	yes	8942	87.69
**Pension insurance**	no	7202	70.63
	yes	2995	29.37

As shown in Table 3, in terms of socioeconomic status, 3134 (30.73%) respondents had an annual income of less than 10,000 yuan, accounting for the highest proportion. Those with annual incomes of 10,000-30,000 yuan were the least, accounting for 18.84% of the respondents. The education level of respondents in the survey was generally low. 5040 (49.43%) respondents reported that they had never attended school and only 996 (9.77%) of them reached education level of senior and above. Among the three main occupational categories given, sample engage in agriculture accounted for 68.46%. Governmental or professional jobs accounted for the lowest percentage of respondents (19.04%). With respect to the social participation, the average score of the item ‘group exercise’ was 4.21 ± 1.97 and the item ‘organized social activities’ was 1.31 ± 0.87. The average score of the item ‘interact with friends’ was 4.14 ± 2.26. Finally, the health status of the respondents, the average score of IADL was 17.98 ± 6.32 and the average score of MMSE was 23.04 ± 8.52.

**Table 3:** Socioeconomic status, social participation and health status of sample

Variables	Category	N	Percentage (%)/Mean ± SD
**Income**	< 10,000 yuan	3134	30.73
	10,000-30,000 yuan	1921	18.84
	30,000-9000 yuan	2700	26.48
	> 90,000 yuan	2442	23.95
**Education**	Illiteracy	5040	49.43
	Primary	3168	31.07
	Junior	993	9.74
	Senior and above	996	9.77
**Occupation**	Agriculture	6981	68.46
	Governmental & professional	1275	12.50
	Commercial & service	1941	19.04
**Group exercise**		10,171	4.21 ± 1.97
**Organized social activities**		10,114	1.31 ± 0.87
**Interact with friends**		10,178	4.14 ± 2.26
**IADL**		10,197	17.98 ± 6.32
**MMSE**		10,197	23.04 ± 8.52

### Results of mediation effect analysis

#### Omnibus mediation effect analysis

First, omnibus effect analysis was conducted. Based on the method proposed by Hayes [43], only when the omnibus total effect test, the omnibus direct effect test and the omnibus mediation effect test were significant, the next step of relative mediation effect analysis could be carried out. As shown in Table 4, with respect to the group exercise, when IADL was the dependent variable, the results of omnibus effect analysis with income or education as independent variables were significant. That was, when income was taken as the independent variable, F value in the omnibus total effect test was 4.94 (*p* = 0.01), indicating that at least one of the there relative total effects was not equal to 0. F value in the omnibus direct effect test was 2.90 (*p* < 0.001), indicating that at least one of the there relative direct effects was not equal to 0. The bootstrap 95% CI of omnibus mediation effect test was (0.002,0.005), 0 was not included. Therefore, it was necessary to make further relative mediation effect analysis. When MMSE was the dependent variable, the omnibus mediation effect test of each independent variable with group exercise as the mediation variable was significant. In terms of the mediation variable ‘interact with friends’, Omnibus test was significant only when education was the independent variable, whether IADL or MMSE was the dependent variable. Finally, when organized social activities was the mediation variable, Except when occupation was the independent variable and IADL was the dependent variable, omnibus test of all variables was significant under other conditions. In addition, every control variable was included in all of the above tests.

**Table 4:** Omnibus effect analysis (F value)

M	X	Y=IADL				Y = MMSE			
		omnibus total effect	omnibus direct effect	bootstrap 95% CI		omnibus total effect	omnibus direct effect	bootstrap 95% CI	
**Group exercise**	**Income**	4.94^**^	2.90^***^	0.002	0.005	14.19^***^	10.20^***^	0.001	0.004
	**Education**	27.53^***^	17.84^***^	0.009	0.016	80.99^***^	68.32^***^	0.007	0.013
	**Occupation**	2.63	1.24	0.003	0.008	28.14^***^	19.68^***^	0.003	0.006
**Interact with friends**	**Income**	5.15^**^	4.34^***^	0	0.002	14.35^***^	11.84^***^	0	0.002
	**Education**	27.46^***^	19.44^***^	0.001	0.005	81.32^***^	71.72^***^	0.001	0.005
	**Occupation**	2.70	4.60^**^	−0.001	0.001	28.17^***^	31.26^***^	0	0.001
organized social activities	income	4.82^**^	3.54^**^	0.001	0.004	14.20^***^	12.75^***^	0.001	0.003
	education	27.16^***^	24.04^***^	0.012	0.023	79.92^***^	76.21^***^	0.007	0.016
	occupation	2.56	0.75	0.007	0.015	28.31^***^	23.00^***^	0.005	0.011

*M* mediation variable, *X* independent variable, *Y* dependent variable, *CI* confidence intervals; the bootstrap 95% CI was for omnibus mediation effect

*p < .05, **p < .01, ***p < .001

#### Relative mediation effect of participating in group exercise

Based on the result of omnibus effect analysis, the test of relative total effect and relative mediation effect was conducted. As shown in Table 5, when IADL was the dependent variable, the variable group exercise has significant relative mediation effect at part of income and education levels on IADL. Specifically, compared with the very-low-income group, the elderly with high income (annual income ≥90,000 yuan) were more likely to improve their physical health by participating in group exercise. The relative mediation effect accounted for 64.11% (a_i_b = 0.24, 95% CI [0.17,0.3]). That was, 64.11% of the improvement in physical health among high-income people was mediated by participation in group exercise. Compared with illiteracy, the elderly with other education level could improve their health by participating in group exercise. The size of relative mediation effect increased when education level promoted. Among the elderly with education of senior and above, 56.30% (a_i_b = 0.62, 95% CI [0.52,0.73]) of the improvement in physical health was mediated by participating in group exercise. When MMSE was the dependent variable, the variable group exercise had significant relative mediation effect at part of income, education and occupation levels on MMSE. Group exercise mediated the improvement of mental health in medium-income and high-income groups. The proportion of relative mediation effect was up to 20.44% (a_i_b = 0.12, 95% CI [0.07,0.17]). Same to the effect on IADL, the size of relative mediation effect on mental health increased when education level promoted. In terms of occupation, compared with the elderly engaged in agriculture, participating in group exercise had a significant relative mediation effect on mental health improvement in both governmental & professional and commercial & service occupational groups. The proportion of relative mediation effect was 16.92% (a_i_b = 0.31, 95% CI [0.23,0.39]) and 28.74% (a_i_b = 0.19, 95% CI [0.13,0.25]), respectively.

**Table 5:** Relative mediation effect of the variable ‘group exercise’

X	c_**i**_	a_**i**_b	bootstrap 95% ***CI***	|a_**i**_b/c_**i**_|	c_**i**_	a_**i**_b	bootstrap 95% ***CI***	|a_**i**_b/c_**i**_|
**income (ref: very low income)**	**Y=IADL**				**Y = MMSE**			
**low income**	−0.06	0.06	(0.01,0.12)	–	−0.01	0.05	(0.01,0.1)	–
**medium income**	−0.08	0.14	(0.08,0.19)	–	0.57^**^	0.12	(0.07,0.17)	20.44%
**high income**	0.37^**^	0.24	(0.17,0.3)	64.11%	1.16^***^	0.20	(0.15,0.27)	17.53%
**education (ref: illiteracy)**								
**primary**	0.98^***^	0.17	(0.12,0.22)	17.07%	2.49^***^	0.14	(0.09,0.18)	5.43%
**junior**	0.86^***^	0.44	(0.34,0.54)	50.66%	2.5^***^	0.35	(0.26,0.44)	14.08%
**senior and above**	1.10^***^	0.62	(0.52,0.73)	56.30%	2.8^***^	0.50	(0.4,0.61)	17.87%
**occupation (ref: agriculture)**								
**governmental & professional**					1.83^***^	0.31	(0.23,0.39)	16.92%
**commercial & service**					0.66^**^	0.19	(0.13,0.25)	28.74%

*X* independent variable, *Y* dependent variable, *ref* reference, *c*_*i*_ the relative total effects of every category in categorical variables on the dependent variable, *a*_*i*_*b* the quantity of relative mediation effect, *|a*_*i*_*b/ c*_*i*_*|* the proportion of relative mediation effect, *CI* confidence interval

*p < .05, **p < .01, ***p < .001

#### Relative mediation effect of interacting with friends

In terms of the mediation variable ‘interact with friends’, the omnibus effect analysis indicated that the mediation effect on the improvement of health status was significant only when the independent variable was education. Referring to Table 6, compared with the elderly who didn’t have any educational experience, the elderly with the education level of primary or junior were more likely to improve the physical health status through interacting with friends. The proportion of relative mediation effect was 24.74% (a_i_b = 0.24, 95% CI [0.16,0.32]) and 33.72% (a_i_b = 0.29, 95% CI [0.16,0.44]), respectively. The relative mediation effect on improvement of physical health was not significant when education level was senior and above (a_i_b = 0.13, 95% CI [0,0.26]). When MMSE was the dependent variable, interacting with friends mediated health status among elderly with different levels of education. The proportion of relative mediation effect was up to 11.20% (a_i_b = 0.28, 95% CI [0.15,0.41]) with the education level of junior.

**Table 6:** Relative mediation effect of the variable ‘interact with friends’

X	c_**i**_	a_**i**_b	bootstrap 95% ***CI***	|a_**i**_b/c_**i**_|	c_**i**_	a_**i**_b	bootstrap 95% ***CI***	|a_**i**_b/c_**i**_|
**education (ref: illiteracy)**	**Y=IADL**				**Y = MMSE**			
**primary**	0.97^***^	0.24	(0.16,0.32)	24.74%	2.49^***^	0.23	(0.16,0.31)	9.24%
**junior**	0.86^***^	0.29	(0.16,0.44)	33.72%	2.50^***^	0.28	(0.15,0.41)	11.20%
**senior and above**	1.10^***^	0.13	(0,0.26)	–	2.80^***^	0.12	(0.01,0.25)	4.29%

*X* independent variable, *ref* reference, *c*_*i*_ the relative total effects of every category in categorical variables on the dependent variable, *a*_*i*_*b* the quantity of relative mediation effect, *|a*_*i*_*b/ c*_*i*_*|* the proportion of relative mediation effect, *CI* confidence interval

*p < .05, **p < .01, ***p < .001

#### Relative mediation effect of participating in organised social activities

As shown in Table 7, when IADL was the dependent variable, the variable organized social activities has significant relative mediation effect at part of income and education levels on IADL. Compared with the very-low-income group, the elderly with high annual income were more likely to have their physical health status improved by participating in organized social activities. The relative mediation effect accounted for 21.20% (a_i_b = 0.08, 95% CI [0.05,0.11]). In terms of education, compared with the elderly group of illiteracy, the elderly with the education level of senior and above were more likely to improve the physical health status through participating in organized social activities, the proportion of relative mediation effect was 21.20% (a_i_b = 0.33, 95% CI [0.26,0.4]).

**Table 7:** Relative mediation effect of the variable ‘organized social activities’

X	c_**i**_	a_**i**_b	bootstrap 95% ***CI***	|a_**i**_b/c_**i**_|	c_**i**_	a_**i**_b	bootstrap 95% ***CI***	|a_**i**_b/c_**i**_|
**income (ref: very low income)**	**Y = IADL**				**Y = MMSE**			
**low-income**	−0.06	− 0.01	(− 0.04,0.01)	–	− 0.01	− 0.01	(− 0.03,0.01)	–
**medium income**	− 0.07	0.03	(0.01,0.06)	–	0.59**	0.02	(0.01,0.04)	4.05%
**high income**	0.37**	0.08	(0.05,0.11)	21.20%	1.15***	0.06	(0.03,0.09)	4.92%
**education (ref: illiteracy)**								
**primary**	0.97***	0.02	(−0.01,0.04)	–	2.48***	0.01	(0,0.03)	–
**junior**	0.85***	0.18	(0.13,0.24)	–	2.47***	0.12	(0.08,0.17)	4.93%
**senior and above**	1.08***	0.33	(0.26,0.4)	21.20%	2.77***	0.22	(0.15,0.28)	7.80%
**occupation (ref: agriculture)**								
**governmental & professional**					1.83***	0.17	(0.12,0.22)	9.16%
**commercial & service**					0.68**	0.09	(0.06,0.12)	13.04%

*X* independent variable, *Y* dependent variable, *ref* reference, *c*_*i*_ the relative total effects of every category in categorical variables on the dependent variable, *a*_*i*_*b* the quantity of relative mediation effect, *|a*_*i*_*b/ c*_*i*_*|* the proportion of relative mediation effect, *CI* confidence interval

*p < .05, **p < .01, ***p < .001

When MMSE was the dependent variable, there existed relative mediation effects at different levels of the three independent variables on MMSE. Specifically, organized social activities mediated the improvement of mental health in medium-income and high-income groups. The size of relative mediation effect accounted for 4.05% (a_i_b = 0.02, 95% CI [0.01,0.04]) and 4.92% (a_i_b = 0.06, 95% CI [0.03,0.09]), respectively. The elderly with education level of junior and above could improve their health by participating in organized social activities when compared with illiteracy. The proportion of relative mediation effect is up to 7.80% (a_i_b = 0.22, 95% CI [0.15,0.28]) with the education level of senior and above. Finally, compared with the elderly engaged in agriculture, the elderly engaged in governmental & professional or commercial & service occupational jobs were more likely to have their mental health status improved through some organized social activities. The relative mediation effect was 13.04% (a_i_b = 0.09, 95% CI [0.06,0.12]) in the commercial & service group and 9.16% (a_i_b = 0.17, 95% CI [0.12,0.22]) in the governmental & professional group.

## Discussion

Based on the sample of the elderly from cross-sectional dataset of CLHLS in 2018, we investigated the association of socioeconomic status on health status and the mediation effect of social participation on this specific association.

First, this study partly validated hypothesis 1, SES was positively related to health status. Specifically, in terms of the effect of income on health status, the results showed that when the income gap between the elderly reached a certain level (more than 80,000 yuan per year in this study), there was a significant difference in physical health status. Differences in mental health status also occurred at smaller income gap, and the trend that the higher the income, the better health condition was presented. High income level was often associated with better nutrition, housing, and medical care, as well as greater health awareness, leading to better health status [45]. In the dimension of education level, significant differences existed in physical and mental health amongst with different education levels. The physical and mental health status of the group with a certain degree of education was better than that of the group without any education. Psychologists believe that people with higher education levels may have greater competence and control over their lives, which in turn improve their social integration and the quality of networks [46]. Jiang et al. [47] found that the education level of the elderly is highly correlated with the prevalence of chronic diseases. Thus, their physical and mental health are better than those with lower education level. Kawachi et al. [48] conducted a review on the potential causal mechanisms linking education and income to health. They also stressed that a causal relationship exists between school education and the improvement in health status, and that increasing the income of the poor leads to an improvement in their health status. In terms of the effect of occupation on health status, our study has not found sufficient evidence to show that occupation has a significant effect on the physical health of the elderly. However, compared with the elderly worked as farmers, the result showed that the elderly engaged in governmental & professional or commercial & service occupational jobs had better mental health status. This difference may be related to their safer and healthier working environment and stronger awareness of health service utilisation [49, 50]. Conversely, influenced by economic stress, low job satisfaction, threat of unemployment, and lack of control over life, an individual is more likely to adopt a passive lifestyle, thereby leading to unhealthy behaviours and depression symptoms [3].

Second, this study validated hypothesis 2 that social participation mediated the effect of socioeconomic status on health status. Specifically, when group exercise was the mediation variable, it mediated more than 60% of the disparity in physical health status and about 17- 20% of the disparity in mental health status due to income gap. As for the disparity in health status caused by different levels of education, participating in group exercise could mediate more than 50% when physical health status was used to compare, and it could mediate 5.43 -17.87% when mental health status was used to compare. Moreover, the greater the gap between the level of education, the higher the proportion of mediation. Group exercise could also mediate some of the differences in mental health status caused by different occupations. However, when we focused on the mediation variable ‘interact with friends’, it mediated only on the path that the education affected the health status of the elderly. With regard to the variable ‘organized social activities’, whether the dependent variable was IADL or MMSE, its relative mediation effect on the health gap caused by income or education level gap was significant at some levels.

When the dependent variable was MMSE, the mediation effect was more sensitive but the mediated proportion was relatively low. Scholars have found that better income condition was often accompanied by more complete infrastructure and exercise equipment, elder people had more opportunities to participate in group exercise activities activities [23]. Regardless of the type of social participation, the degree of activity participation has a significant positive correlation with the physical and mental health of the elderly, indicating that social participation can significantly improve their health status [27, 51]. A series of studies have shown that relatively close social relations of the elderly are important in protecting them against cognitive decline [52, 53], dementia, and memory loss [54]. Activity theory holds that social participation can buffer the negative effects of ageing on mental health by meeting the psychological and social needs of the elderly [55]. According to this theory, the elderly find their identity and meaning through their social roles, thereby energising their lives. And the effect is easier to achieve if they have good economic conditions and a high level of education.

## Conclusion

Through this study, we mainly found that the SES, including relatively large income gap, different education levels and occupational categories could indeed have a significant effect on the physical and mental health of the elderly, and the reason why this effect existed could be partly explained by the mediation effect of social participation. This result suggested that, on the one hand, policymakers should pay more attention to the level of family income of the elderly and their economic security within the family. More opportunities should be given for the elderly to seek self-learning and advancement, such as continuing education. At the same time, pay attention to the different occupation categories of the elderly before retirement, and give different levels of security policies. On the other hand, in order to improve the physical and mental health of the elderly, policymakers should give more encouragement and support to the social participation of the elderly, including improving the infrastructure of public areas, advocating collective exercise activities, and guiding the elderly to participate in group cultural and recreational activities. Especially for the elderly with lower socioeconomic status, the adverse effects of lower socioeconomic status on health status can be mitigated by supporting them to participate in more social activities.

## Data Availability

The CLHLS datasets are publicly available at the National Archive of Computerized Data on Aging, University of Michigan (http://www.icpsr.umich.edu/icpsrweb/NACDA/studies/36179). Researchers can obtain these data after submitting a data use agreement to the CLHLS team.

## Abbreviations

CLHLS: Chinese Longitudinal Healthy Longevity Survey
IADL: Instrumental Activities of Daily Living Scale
MMSE: Minimum Mental State Examination
GE: Group exercise
IF: Interact with friends
OSA: Organized social activities
SE: Standard error
CI: Confidence interval
